# Salivary endocrine response following a maximal incremental cycling protocol with local vibration

**DOI:** 10.1371/journal.pone.0238051

**Published:** 2020-09-11

**Authors:** Monèm Jemni, Michel Marina, Anne Delextrat, Amy Tanner, Fabien A. Basset, Yaodong Gu, Qiuli Hu, Huiyu Zhou, Bessem Mkaouer, Ferman Konukman

**Affiliations:** 1 Faculty of Sports Science, Ningbo University, Zhejiang, China; 2 The University of Cambridge—Institute of Continuing Education, Cambridge, United Kingdom; 3 INEFC, Barcelona, Spain; 4 Department of Sport, Health Sciences and Social Work, Oxford Brookes University, Oxford, United Kingdom; 5 School of Life and Medical Sciences, University of Hertfordshire, Hatfield, United Kingdom; 6 School of Human Kinetics and Recreation, Memorial University of Newfoundland, St. John's, Canada; 7 Higher Institute of Sport and Physical Education of Ksar Saïd, Manouba University, Manouba, Tunisia; 8 Sport Science Program, College of Arts and Science, Qatar University, Doha, Qatar; São Paulo State University (UNESP), BRAZIL

## Abstract

The aim of this study was to compare the effects of vibration (Vib versus noVib) during a maximal graded cycling exercise on hormonal response, precisely on cortisol (C) and testosterone (T). Twelve active males (25 ± 5yrs; 181 ± 5cm; 80.7 ± 11.1kg) randomly performed two maximal incremental cycling tests on two separate days and at the same time of the day (09:00). The protocol consisted of incremental steps of 3 min duration performed on a PowerBIKE^TM^ that induces vibration cycling. The study was a repeated measures design and participants performed the test with and without vibration. Gas exchange and heart rate (HR) were continuously assessed and blood lactate (Bla) was recorded at the end of each incremental stage. Saliva samples were collected before and immediately after the test, and analysed for (C) and (T).

The results show that C and T increased in both cycling conditions; however, the C’s magnitude of change was significantly higher by 83% after Vib cycling in comparison to the no Vib (*p* = 0.014), whereas the T’s magnitude of change were not statistically different between trials (*p* = 0.715). Vibration induced a decrease of the T/C ratio (*p* = 0.046) but no significant changes were observed following noVib (*p* = 0.476). As a conclusion, the investigation suggests that adding mechanical vibration to cycling may potentiate a catabolic exercise-induced state, which could have potential clinical implications in rehabilitation and injury treatment. Sport experts should take this message home to carefully plan the recovery process and time during training and competitions.

## Introduction

Local body vibration (LBV) during dynamic activity has only recently been applied to cycling exercise. Early and recent research on vibration cycling has reported a significant decrease in exercise duration compared to cycling-only trials [1]. However, more recent and meticulously controlled trials have indicated no differences in cardiorespiratory and metabolic variables between vibration cycling and cycling-only exercises, except a higher ventilation in favour of the vibration cycling [2]. These recent finding did not confirm prior studies who suggested higher rates of oxygen uptake, possibly due to vibration-induced activation of afferent neurons causing contraction of inactive muscle fibres [1, 3]. Note that there is a difference between whole body vibration exercise/training using platforms in comparison to vibration cycling which is the context of this study. Whole body vibration exercise showed consistent significant improvement of muscles’ viscoelasticity and flexibility [4] whereas studies investigating its effect on strength are not unanimous, with some showing positive effects [4–6] and others showing the opposite [7] or no effect [4, 8].

Most of the studies examining the hormonal responses induced by vibration during training have been conducted on resistance training and have reported conflicting results, such as an elevation of the plasma testosterone [9], plasma cortisol [10] or no change [11–13]. These mixed results might stem from an insufficient exercise stimulus to elicit a change in hormonal response [14]. Therefore, it seems rational to investigate the hormonal response during high-intensity aerobic exercise. This study is indeed the first of a series of investigations that highlight several effects of different exercise regimes on the hormonal regulation with a particular focus on testosterone and cortisol.

During acute stress, activation of the sympathetic nervous system occurs, with the release of catecholamines and concomitantly, the hypothalamic-pituitary-adrenal (HPA) axis is stimulated and cortisol is released [15]. Cortisol plays a permissive role on catecholamines and glucagon in stimulation of gluconeogenesis and mobilisation of free fatty acids to initiate glucose maintenance [16], this is particularly important in response to stress and may reflect the metabolic demand of an exercise bout [17]. Furthermore, short term, acute stress has also been shown to increase circulating levels of testosterone [18]. Previous work has established that an incremental test to exhaustion elicits an increase in salivary cortisol [19, 20] and correlates with blood lactate measurements [19]; with suggestion that lactate may activate chemoreceptors in the working muscles and stimulates the HPA axis [21]. Furthermore, an increase in salivary cortisol and testosterone was observed after a short duration high intensity cycling bout [22, 23]. Consistently, studies looking at aerobic exercise have demonstrated increases in cortisol and testosterone; whereas, there is considerable variability in results following resistance based exercise for cortisol and power based exercise for testosterone [24]. As such, high-intensity exercises, such as speed endurance maintenance and speed endurance production lead to a significant acute increase in circulating cortisol levels [25]. However, the same authors did not confirm a long-term trend but rather a slow decrease of the cortisol production after repeated sprints training. Testosterone has been shown to increase few minutes post adequately stimulating resistance exercise. According to (Kraemer and Ratamess 2005 [26], high volume protocols, moderate to high in intensity, and exercise regimes that incorporate short rest intervals and involve a large muscle mass, tend to produce the greatest acute hormonal elevations (testosterone, Growth Hormone and Cortisol) when compared to low-volume, high-intensity protocols incorporating long rest intervals.

A recent study showed that vibration-induced cycling did not increase energy demands [2]. However, the question that remains unanswered is what are the effects of vibration on cortisol and other markers of metabolic stress during maximal exercise. Therefore, the aim of the study was to assess the impact of added vibration to cycling using an incremental exercise test on the salivary endocrine response compared to normal cycling. Our hypothesis states that added vibration stimuli to cycling would increase the body’s endocrine response, in particular, the cortisol.

## Methods

### Participants

A call for participation in a research project was publicized across the University of Greenwich (London, UK) sports clubs via the social media network in June 2014. The inclusion criteria were: males only, recreationally active by performing at least three hours/week of sport for at least one year, age range between 18–28 years old, not suffering from any cardiorespiratory diseases, no family history of similar diseases. Exclusion criteria were: females, inactive and/or sedentary males, younger than 18 or older than 28 years old, suffering from any cardiorespiratory diseases, family history of similar diseases.

Twelve males agreed to take part in the study. Most of them were students but also staff members working across the campus. All have been seen by the main investigator and verbally briefed about the study and the risks associated. Written information and instructions were given to them to take away and written consent for participation was obtained from each participant. The study was approved by the University of Greenwich ethics committee (Ref: UoG-EC-FSE-SS-J-L-35-20/1/2014) and the research took place at the Faculty of Science and Engineering labs between July and September 2014.

Participants’ anthropometrics characteristics were: age 25 ± 5 yrs; height 181 ± 5cm and weight 80.7 ± 11.9kg.

### Testing procedure

Each participant performed two maximal incremental cycling exercise tests, one with vibration (Vib) and one without vibration (noVib) in a randomised order, on separate days and at similar times of the day (09:00). Tests were performed at the same time of the day, with a recovery period of 72 hours in between. Participants continued their habitual training regimen during the study period; however, they were asked to refrain from eating two hours prior to all trials, and from strenuous exercise, caffeine and alcohol consumption in the 24 hours before each trial. All participants were invited for a screening questionnaire as well as a familiarization session and to randomise the trials a few days prior the testing day.

**Fig 1:**
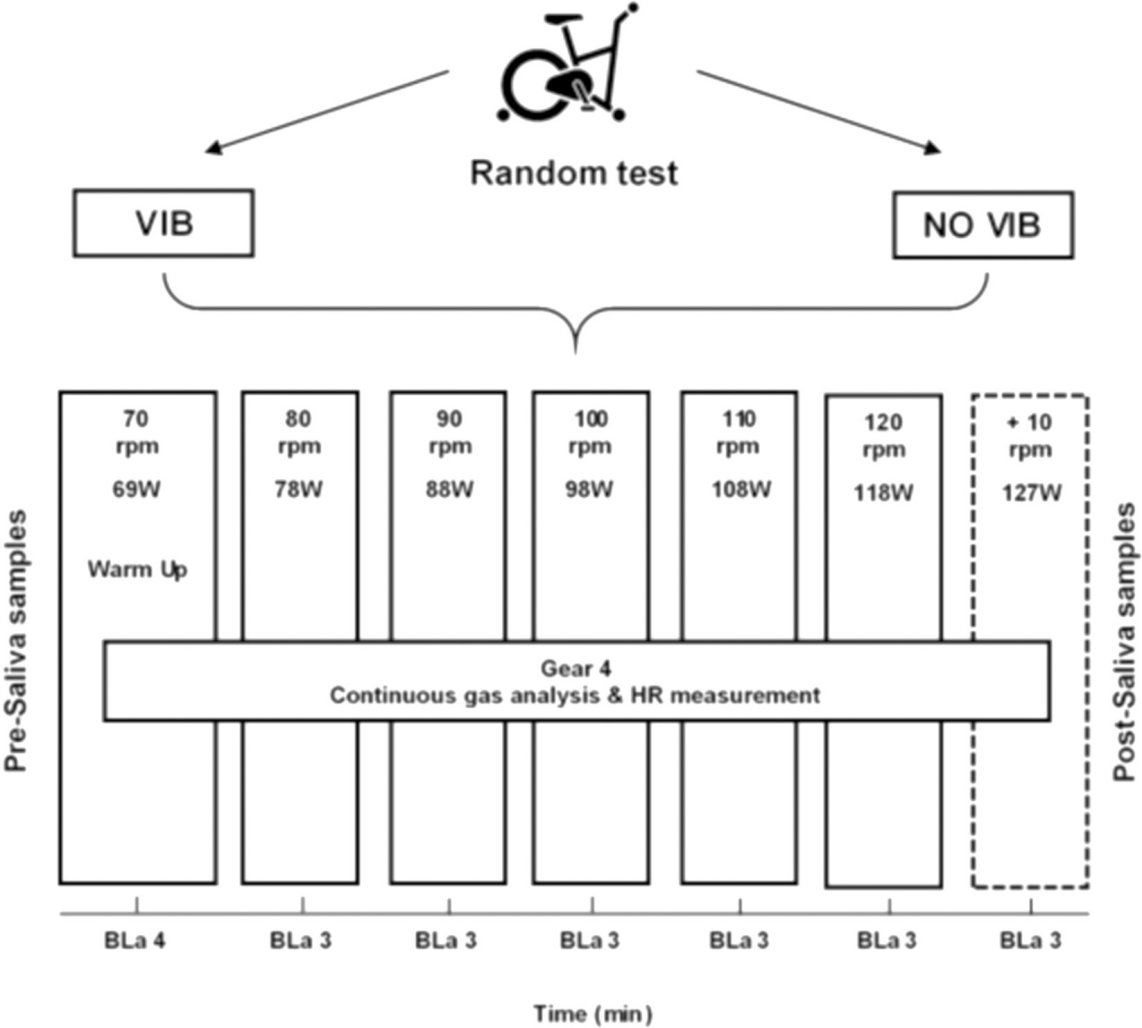
Flow chart of the experimental study. Graded exercise cycling tests to the maximum on PowerBike.

All exercise tests were performed on the PowerBIKE (Power Plate, Netherlands), a stationary bike that induces Vib cycling or normal cycling. The protocol was a speed-based and similar to a previously described study [27]. After a 4 minute warm up at 70 RPM at (4th gear) cadence was increased 10 RPM every three minutes until volitional exhaustion (Fig 1). The PowerBIKE was set according to participants’ anatomy: the height of the seat, distance between the handle bar and the seat, seat to centre of the crank, handle bar to the centre of the crank and handle bar to floor, recorded and applied identically at each trial.

### Physiological measurements

Gas exchange was continuously assessed with an online gas analyzer Vacumed Metabolic Measurement System (Metamax, Cortex, Germany) monitored by a TurboFit software, V. 5.0 (USA). A five μL blood sample was collected from the fingertip at rest and during the last 30 seconds of each exercise stage. Samples were analysed for blood lactate (BLa, mmol•l^-1^) concentration using a lactate analyser (Biosen EKF diagnostic, Germany). Heart rate (HR, beats•min^-1^) was continuously monitored using a HR monitor (Polar, Finland) and averaged for the last 30 seconds of each stage; maximal HR was recorded at the end of each test (HR_max_).

### Saliva collection and cortisol and testosterone measurement

Saliva samples were collected pre-exercise and immediately post-exercise during each trial. Hence, each participant had four saliva samples. Each subject was required to attend his tests sessions at the same time of the day to avoid diurnal variation. Salivary measurement was chosen as there is evidence that salivary cortisol and teststerone levels offer a more sensitive assessment of the response to exercise than changes in blood concentration [22, 23]. Participants were required to stop drinking ten minutes before each sample collection to avoid dilution. Participants provided a stimulated saliva sample into a sterile container, with Parafilm to chew on to increase flow, since cortisol and testosterone are unaffected by saliva flow rate [28, 29]. Prior to collection participants were instructed to chew for one minute before swallowing any saliva in the oral cavity. The sampling time was three minutes to allow collection of a sufficient saliva volume. Samples were centrifuged at 3000rpm, divided into four aliquots and stored at -20°C. Saliva was analysed for cortisol and testosterone with a commercially available ELISA kits (Salimetrics, State College, PA, USA). The sensitivity of the kit was 0.029 ng•ml^-1^ for cortisol and 1 pg•ml^-1^ for testosterone. The mean intra assay coefficients of variation were 8.3% for cortisol and 8.6% testosterone for duplicate samples.

### Statistical analysis

The Shapiro-Wilk test confirmed the normal distribution of the data (*p*: 95% CI [0.422, 0.681]) enabling parametric analysis of the variables. Data is presented as mean and standard deviation. Cortisol, testosterone, HR and BLa differences, between pre-exercise and post-exercise as well as between Vib and noVib cycling conditions were assessed with a Two-Way (2 trials x 2 conditions) analysis of variance for repeated measures (ANOVA). The Greenhouse-Geisser’s correction factor was applied if the sphericity test for proportionality of the dependent variable was significant (*p* < 0.05). Bonferroni *post hoc* adjustments were used for multiple comparisons and partial eta squared (η2) was used to report effect size.

Note that only nine participants were included in the hormonal analysis because of technical errors. The magnitude of changes induced by the cycling tests were calculated for cortisol, testosterone and T/C ratio by subtracting post-exercise values from pre-exercise. These were thereafter expressed as raw differences as well as in percentage relative to baseline (relative increments). Cohen’s d test with Hedges’ g correction were used to report effect size when pairwise comparing (Vib/NoVib). Statistical analysis was undertaken using SPSS statistics package software version 20 for Windows® (IBM, NY, USA). Statistical significance was accepted at *p* ≤ 0.05.

## Results

### Heart rate

Resting HR was not different before both trials (*p =* 0.98). Maximal values (HR_max_) did not statistically differ between the Vib and the no Vib trials (177 ± 13 and 181 ± 16 BPM respectively) at the end of the tests.

### Maximal oxygen uptake (${\dot{V}O}_{2\max}$)

${\dot{V}O}_{2\max}$ did not significantly differ (*p* = 0.1631; *d* = 0.60) between Vib and no Vib trials (34.32 ± 9.7 and 40.11 ± 9.49 ml•min^-1^•kg^-1^, respectively) although they were reached at an average of 100 RPM with Vib and 120 RPM without Vib (98W after 13 min cycling and 118W after 19 min cycling respectively).

### Blood lactate (BL)

The maximal BLa concentration (BLa_max_) measured at the end of each trial was significantly higher (*p* < 0.05; *d* = 0.87) after the Vib trial (14.05 ± 2.86 mmol•l^-1^) compared to no Vib (11.31 ± 3.44 mmol•l^-1^)

### Salivary cortisol

**Fig 2:**
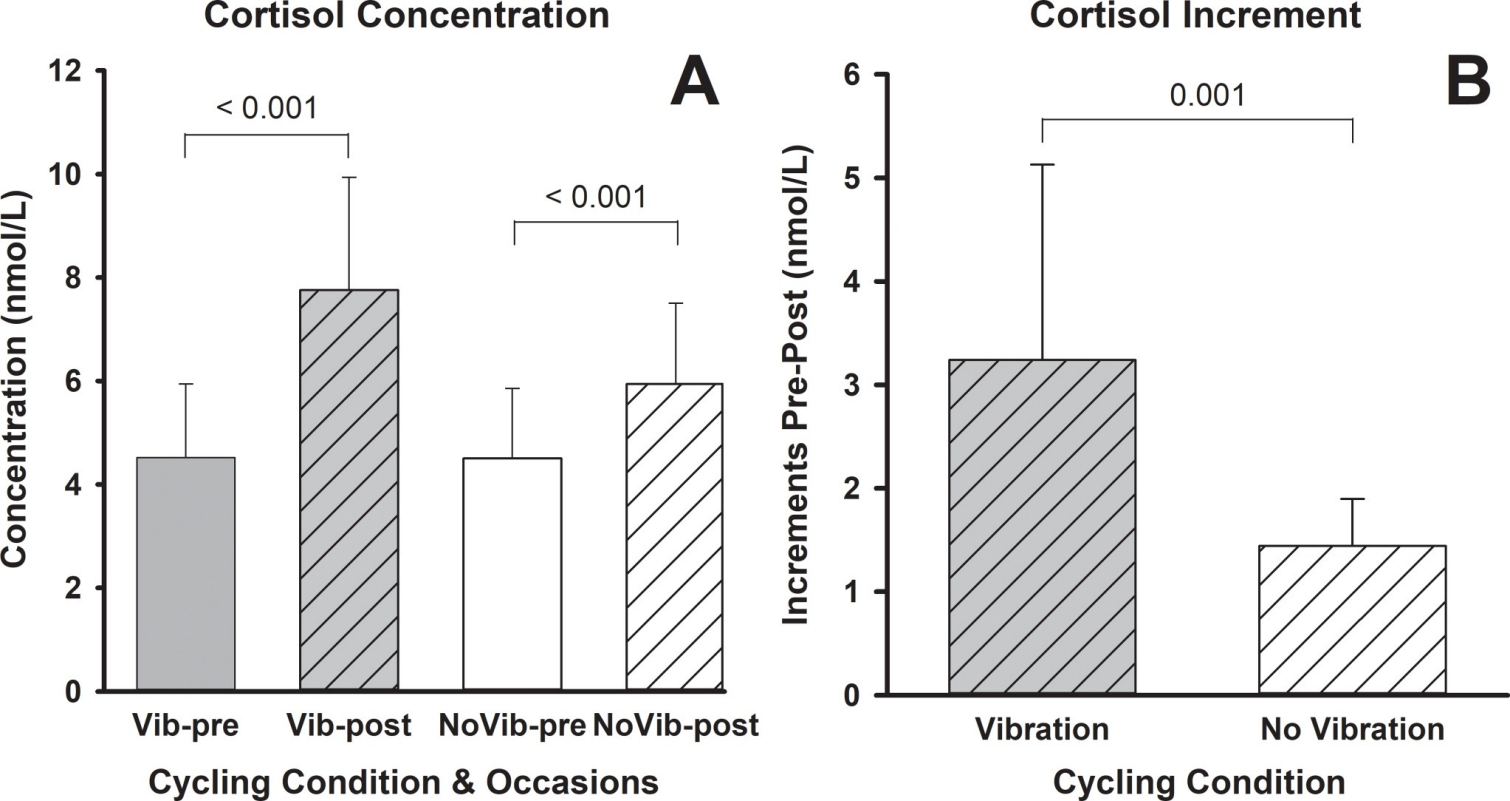
Salivary cortisol pre- and post-maximal graded exercise with and without vibration (A) and their respective magnitude of change (B). S: (*p*≤0.05).

Baseline cortisol levels were not significantly different before Vib and noVib (*p* = 0.980). ANOVA analysis revealed that the cortisol level increased significantly after both cycling tests (Vib and noVib) (Table 1) (Fig 2). Moreover, an interaction between the trial and vibration factors suggest a differentiated pattern of change when both factors are combined. The increase in cortisol concentration was significantly higher after the Vib cycling test in comparison to the no Vib. Cortisol increased by 83% after the Vib trial (Fig 2A). Individual comparative tests confirmed this observation for both, the absolute increment (*p* = 0.014) (Fig 1B; *p* = 0.022) and relative increment with respect to baseline condition (Fig 4B).

**Table 1:** Statistical analysis of the raw data for the cortisol, testosterone and their ratio pre- and post-cycling with or without vibration.

Variable	Effect	*F*	*df*	*P*	η2	Post-hoc	*P*
Cortisol	Oc x Vb	9.91	1, 8	0.014	0.55	Vb: O_c2_ > O_c1_	0.001
						nVb: O_c2_ > O_c1_	0.001
	Oc	43.09	1, 8	0.001	0.84	O_c2_ > O_c1_	0.001
	Vb	1.54	1, 8	0.240	0.16		
Testosterone	Oc x Vb	3.16	1, 8	0.113	0.28		
	Oc	81.52	1, 8	0.001	0.91	O_c2_ > O_c1_	0.001
	Vb	0.04	1, 8	0.839	0.01		
Ratio T/C	Oc x Vb	21.07	1, 8	0.002	0.73	Vb: O_c2_ > O_c1_	0.046
						nVb: O_c2_ ≈ O_c1_	0.476
	Oc	1.38	1, 8	0.274	0.15		
	Vb	0.76	1, 8	0.408	0.09		

Oc: occasion; Vb: vibration; nVb: no vibration

### Salivary testosterone

**Fig 3:**
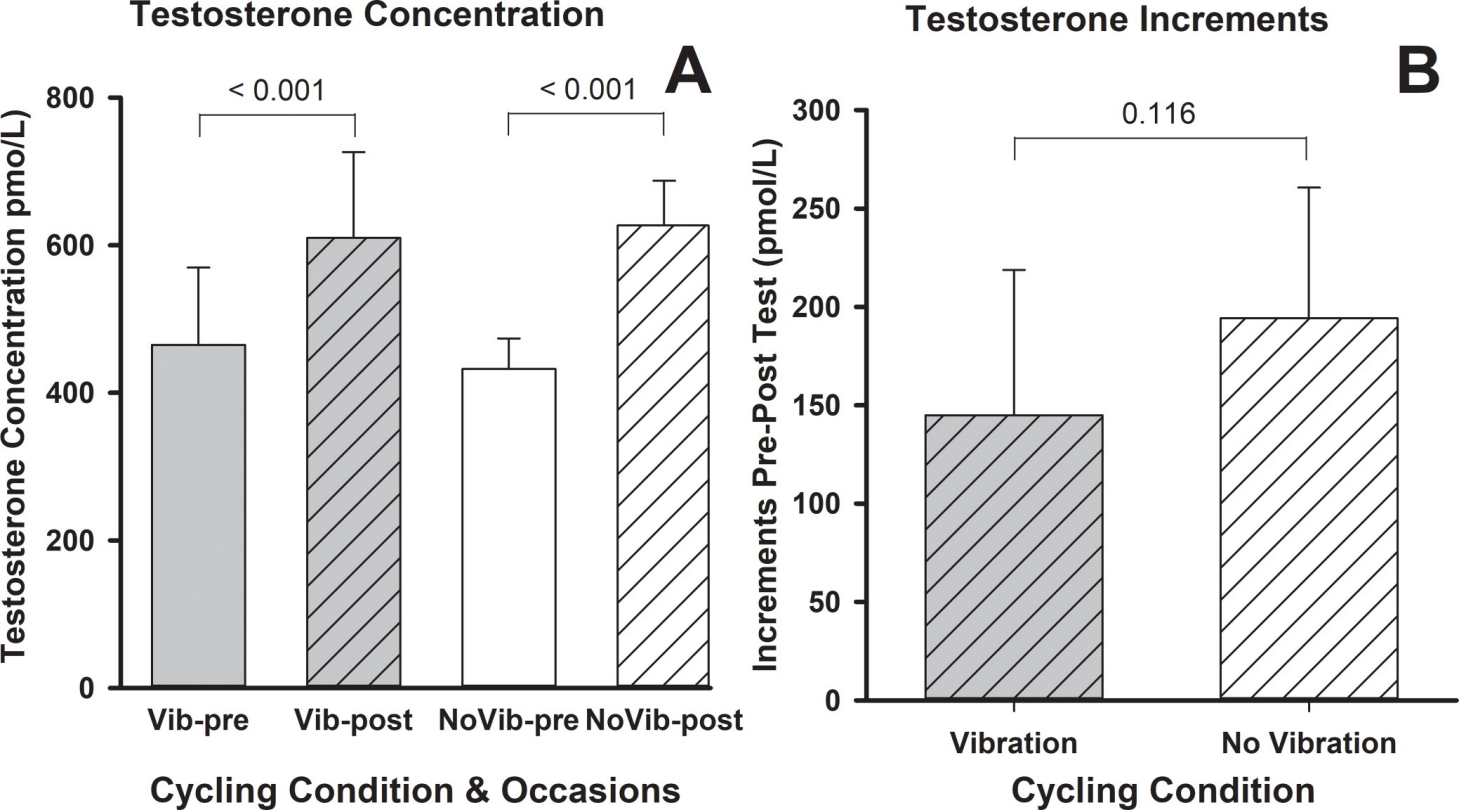
Salivary testosterone pre- and post-maximal graded exercise with and without vibration (A) and their respective magnitude of change (B). S: (*p*≤0.05).

Baseline testosterone levels were not significantly different before Vib and noVib (*p* = 0.392). Salivary testosterone increased by 29% after Vib and 56% after noVib cycling test (Fig 3). Statistical analysis revealed that testosterone level increased significantly after both cycling tests (Vib and noVib) (Table 1); however, there was no statistical difference in magnitude of change between trials (*p* = 0.715) (Fig 3B).

**Fig 4:**
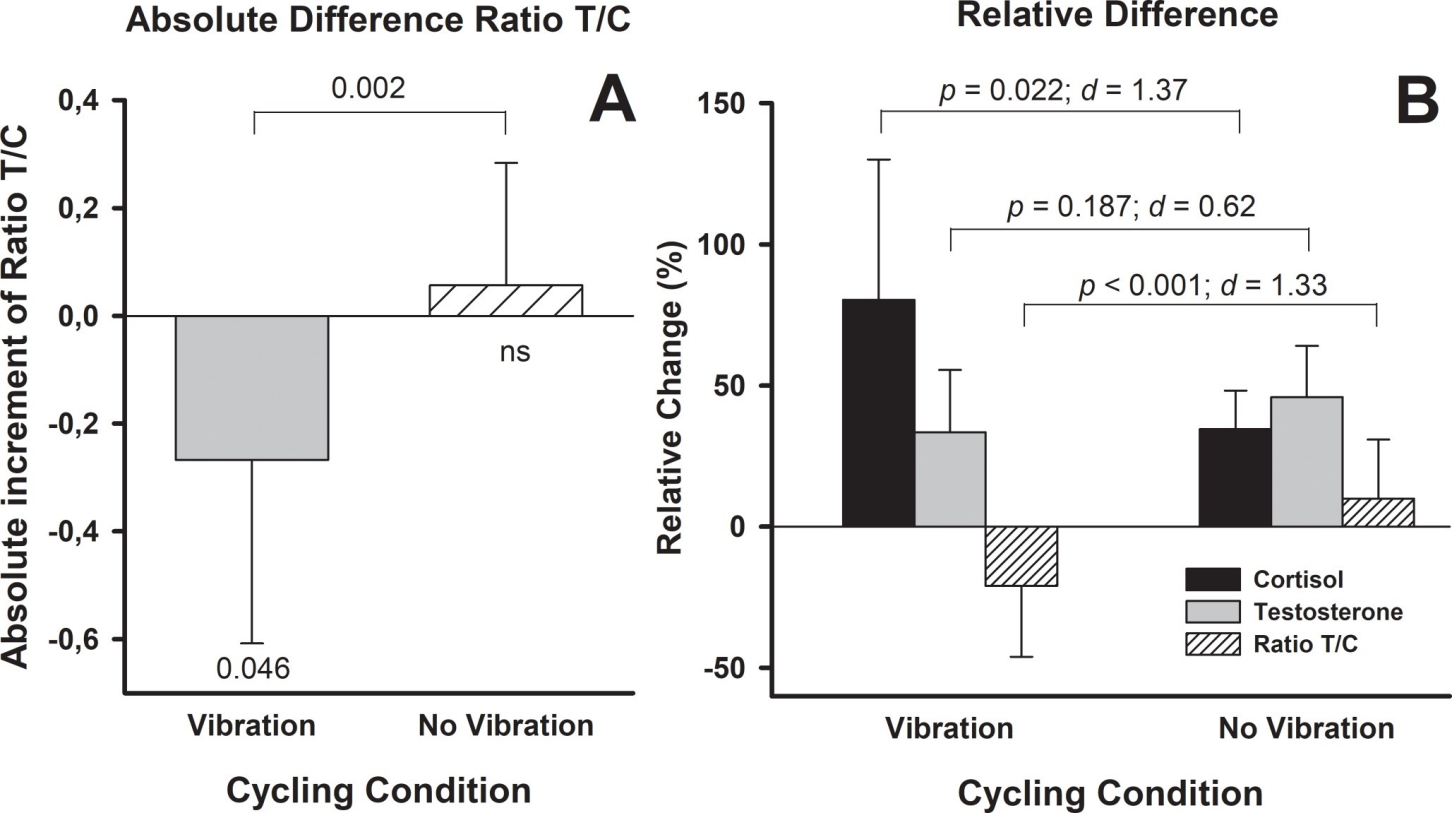
Absolute increments of the testosterone and cortisol ratios induced by the cycling graded exercise with and without vibration (A). Pairwise comparisons of the relative differences with respect to baseline (expressed in %) between the two cycling conditions (B).

Paired tests confirmed that the magnitude of the absolute (*p* = 0.116) and the relative increments with respect to baseline (*p* = 0.187) were similar after the two cycling tests (Fig 4B).

### Testosterone/cortisol (T/C) ratio

Baseline T/C ratios were not significantly different before Vib and noVib (*p* = 0.825). A signifcant main effect confirmed that Vib induced a decrease of the T/C ratio (*p* = 0.046) whereas no significant changes were observed following noVib (*p* = 0.476) (Table 1; Fig 4A).

There was a significant difference in the absolute (*p* < 0.002) and relative (*p* ≤ 0.001) change in T/C ratio following the Vib trial compared to noVib (Fig 4).

## Discussion

The main hypothesis of this study was that added vibration stimuli to cycling would increase the salivary endocrine response, in particular, the cortisol level. Larger and significant cortisol increase associated to a larger and significant T/C ratio decrease were noticed in the Vib trial compared to noVib. While some authors have reported decrease in constant-load cycling duration with the addition of a vibratory stimulus [1] others did not identified this trend [3]. Exercising with vibration has been hypothesised to recruit more motor neurons [30], and it has been suggested that full activation of the muscle may lead to a quicker motor unit fatigue [31, 32] and as a result contribute to an earlier onset of fatigue in Vibration exertions [33]. Bongiovanni and al. [34] argued that the decreased ability to generate high firing rates in high threshold motor units may cause the inability to sustain exercise. Another reason for this difference may be a higher energy demand with the addition of Vib, contributing to an increase in the ATP hydrolysis [35]. However, this study did not show any difference in the ${\dot{V}O}_{2\;\max}$, hence re-enforces our latest findings that adding vibration to cycling did not induce a greater cardiorespiratory response compared to normal cycling [2]. Nonetheless, we acknowledge the non-negligible difference in ${\dot{V}O}_{2\max}$ between the trials (more than—15%). A potential protocol effect could have been masked by not only the inter-participants' variability but also by the limited sample size (only 12 participants). The participants in the present study were recreationally active, undertaking a mixture of aerobic and weight training on average of three days per week; therefore, they do not represent an elite or competitive athlete population and as a consequence, these results should not be translated to a more highly trained cohort and neither to unfit or unhealthy indivuduals. Yet, the results could have some implications for a much wider and general population whose objective is to increase fitness for various reasons (e.g., quality of life). On the other side, one could question the higher BLa following the Vib trial compared to no Vib; this could undeniably be explained by the fact that the participants have perceived the Vib cycling stronger than no Vib as it was previously demonstrated by Filingerie et al in 2012 [27].

The results of this study show an increase in cortisol and testosterone in both trials. Similar high-intensity protocols have also triggered increased cortisol levels following acute exercise bouts [19, 22, 36]. Nevertheless, we do not know about the existence of previous research focused on cortisol levels in response to cycling with vibration. The present study supports observations of an increase in cortisol with vibration added up to resistance exercise [10]. Some scientists have hypothesised an increase in cortisol to be linked with blood lactate levels [19, 21, 37]. Given the observed higher blood lactate of the Vib trial in our study, this may have activated the HPA axis and led to an increase in cortisol concentration.

Salivary testosterone increased in both trials of the present study. It has been hypothesised that possible mechanisms for an increase in testosterone, as a result of exercise, include increased production by sympathetic stimulation of the testes [38]. Furthermore, activation of the sympathetic nervous system and increased lactate accumulation may have contributed to the increase in testosterone concentration, although supporting evidence is limited to rats [39]. Protein binding affinity of the testosterone may be affected by changes in PH and temperature elicited by exercise, this in turn may lead to a higher free proportion of cortisol and testosterone in the blood and increased levels in saliva [40, 41]. However, a more recent study showed no binding affinity of testosterone changes after endurance exercise [38] and further research is surely required.

Only very few studies have examined the response to vibration on testosterone levels. Bosco et al. [9] demonstrated an increase in plasma testosterone following ten 60-second bouts on a vibration platform. However, as the present study showed no significant additive effect of vibration on salivary testosterone levels, hence these findings cannot be supported. It is worth to mention that Bosco and his colleagues [4] have used a whole body vibration whereas we only used localised vibration induced by the cycling gear through the lower limbs. The number of stimulated neuromuscular units could have made this difference.

There is suggestion that testosterone may also contribute to muscular repair and growth in response to training, however its role in this process has not been confirmed [42]. Owing to the absence of a bigger increment of testosterone as a result of vibration in the present study, and limited evidence to form a clear consensus, further research in this area is also warranted. Should we admit that testosterone is just less sensitive to small changes in demand?

The T/C ratio showed a trend towards a decrease in the Vib trial, this is likely to be due to the higher increase in cortisol after Vib. This decrease suggests that following Vib induced cycling the body was in a more catabolic state and perhaps a higher acute response was initiated with the addition of Vib. In support, a meta-analysis examining T/C in response to aerobic exercise showed a consistent decrease in the ratio which was primarily due to the magnitude of the salivary cortisol response [24]. However, the T/C ratio has proven to be more useful when considering the link with overreaching and overtraining; including absolute values, comparison of consecutive measurements during a season or changes in relation to baseline. Monitoring the T/C ratio in athletes has the potential to be used as a tool to diagnose overtraining syndrome while taking into account other clinical measurements [43]. Previous research has recognised that the mode of exercise and volume of stimulus is important in the hormonal response [17, 37]; therefore, it is currently difficult to make valid comparisons between vibration studies, given the use of many different protocols. Nonetheless, there is a increasing clinical interest about vibration mainly following the confirmed positive effects on muscles’ viscoelasticity [4]. Combining vibration and exercise could see further applications in the medical sector where exercise alone has been suggested to prevent and to treat/rehabilitate certain conditions.

The study has few methodological considerations/limitations. Firstly, it has been carried out with recreational active subjects allowing statistical inference to a normal active and healthy population. These results cannot be extrapolated to neither elite oriented athletes nor unfit or unhealthy individuals, as it was not the aim of the present investigation. In addition, seeing the small sample size, it cannot be considered representative of a larger population. Secondly, saliva samples were taken only once before and just after the two trials (Vib and noVib cycling), while mostly the peak salivary cortisol and testosterone levels occur immediately post exercise, they have been shown to peak later in some individuals as a result of intermittent exercise [17]. Although our protocol was based on only one single long cycling bout, it could be worth investigating post-exercise peak hormone values in future studies. However, we have tried to double limit the C and T circannual variations by collecting the samples not only at the same time of the day for each participant but also within a minimum time (12 weeks). A recent study has indeed demonstrated that C, T and T/C incur significant circannual variations in professional footballer players, with the cortisol increasing during winter (February) while testosterone and T/C peak in the summer months (July) [44]. Lastly, it is important to state that the increment between the test stages was not the same in comparison to the study we published in 2019 [2]. Two different bikes were used in Jemni et al, 2019 (Lode Corvical and PowerBike), where 1 watt increment was equivalent to 18 ml/min. However, in this study the bike was the same, i.e the PowerBike, where 1 watt increment provoked an equivalent (27.5 ml/min). We presume that the higher value was due not only to the ergonomics difference between the bikes but also to the fitness levels of the different studies’ cohorts. Jemni 2019 participants were moderately trained male subjects with six to eight hours of training per week (amongst them few cyclists), whereas this study involved few students and staff members from the university campus who were slightly less active.

The results discussed in the present investigation justify further research focused on vibration exertion to better understand the complex relationship between hormonal, metabolic, cardiovascular, respiratory and neurophysiological markers. Moreover, biomechanical and electromyographic assessments could help to better understand the pattern of these markers and their reciprocal influence with metabolic cost and muscle activation.

Finally, the fact that vibration cycling induced a higher acute catabolism in this investigation could lead to further practical implications either in the exercise training context or in the clinical sector where such state is required. Making the competition weight in certain sports or purposely elevating the catabolism state in certain health conditions could be of interest.

## Conclusion

The main aim of this study was to examine the salivary endocrine response to vibration cycling exercise. We were expecting a higher cortisol response compared to the no vibration condition. Overall, it appears that a Vib cycling trial elicited a greater cortisol increase. Therefore, the null hypothesis is not accepted.

Cortisol increased dramatically after the Vib cycling and was significantly higher in comparison to the no Vib. Salivary testosterone has also increased after Vib cycling; however, there was no statistical difference in the magnitude of change between the trials. Vib induced a significant decrease of the T/C ratio whereas no significant changes were observed following noVib.

The study suggest that adding mechanical vibration to cycling may potentiate a catabolic exercise-induced state. Sport experts and mainly cyclists should take this message home to carefully plan the recovery process and time during training and competitions. The overall results could have potential clinical implications for older individuals and those undergoing rehabilitation from illness and injury. Future research should investigate the longer-term response to cycling exercise with added vibration.
